# Transferrin Receptor Marks a Foxp3-Low Treg-like Inflammatory T Cell Subset Associated with Disease Severity in HAM/TSP

**DOI:** 10.3390/pathogens15040450

**Published:** 2026-04-21

**Authors:** Shinsuke Nakajima, Masaki Hino, Norihiro Takenouchi, Yoshihisa Yamano, Makoto Yamagishi, Tokifumi Odaka, Fhahira Rizkhika Admadiani, Cecile Faye, Kaoru Uchimaru, Jun-Ichi Fujisawa, Kazu Okuma

**Affiliations:** 1 Department of Microbiology, Faculty of Medicine, Kansai Medical University, Hirakata 573-1010, Japan; 2 Department of Rare Diseases Research, Institute of Medical Science, St. Marianna University School of Medicine, Kawasaki 216-8511, Japan; 3 Laboratory of Viral Oncology and Genomics, Department of Computational Biology and Medical Sciences, Graduate School of Frontier Sciences, The University of Tokyo, Tokyo 108-8639, Japan; 4 Central Research Center, Institute of Biomedical Science, Kansai Medical University, Hirakata 573-1010, Japan; 5 Laboratory of Tumor Cell Biology, Department of Computational Biology and Medical Sciences, Graduate School of Frontier Sciences, University of Tokyo, Tokyo 108-8639, Japan; 6 Faculty of Medicine, Showa Medical University, Tokyo 142-8555, Japan

**Keywords:** HTLV-1, HAM, transferrin receptor, regulatory T-cell

## Abstract

Human T-cell leukemia virus type 1 (HTLV-1)-associated myelopathy/tropical spastic paraparesis (HAM/TSP) is a chronic inflammatory disease driven by HTLV-1-infected CD4^+^ T cells; however, the phenotypic and functional characteristics of disease-associated T-cell subsets remain incompletely understood. We analyzed samples using flow cytometry (*n* = 3–5 per group) and RNA-seq (*n* = 13), focusing on CADM1^high^CD4^+^ T cells enriched for HTLV-1-infected cells to evaluate a transferrin receptor (TfR)-expressing subset. TfR^+^CADM1^high^CD4^+^ T cells were detected in both asymptomatic carriers and patients with HAM, but their frequency among CD4^+^ T cells was higher in HAM patients. These cells exhibited a Treg-like phenotype with higher Foxp3 and CTLA-4 expression than TfR^−^ cells and showed increased Ki-67 positivity, consistent with proliferation. Despite this phenotype, they produced interferon-γ, indicating inflammatory potential, while Foxp3 expression was lower in HAM patients than in asymptomatic carriers, suggesting a more inflammatory phenotype. Furthermore, TfR transcript levels (RNA-seq TPM) correlated with clinical indicators of disease activity, including neopterin and CXCL10 protein levels, and the Osame motor disability score. Collectively, these findings suggest that TfR identifies a proliferative, Foxp3-low, Treg-like inflammatory CD4^+^ T-cell subset that is associated with disease activity in HAM.

## 1. Introduction

Human T-cell leukemia virus type 1 (HTLV-1) was the first pathogenic human retrovirus to be identified [1,2]. After a prolonged latency period of several years to decades, HTLV-1 infection can result in adult T-cell leukemia/lymphoma (ATLL) [3,4] or HTLV-1-associated myelopathy/tropical spastic paraparesis (HAM/TSP) [5,6]. In patients with HAM, HTLV-1-infected CD4^+^ T cells, as well as cytotoxic CD8^+^ T cells, that recognize the viral Tax antigen infiltrate the spinal cord and produce proinflammatory cytokines, thereby contributing to chronic neuroinflammation and tissue damage [7,8]. A high proviral load in peripheral blood mononuclear cells (PBMCs) is strongly associated with disease severity and progression in HAM [9], highlighting the importance of infected CD4^+^ T cells in disease pathogenesis.

Cell adhesion molecule 1 (CADM1) is commonly used as a surface marker to broadly identify HTLV-1-infected CD4^+^ T cells; however, it is not entirely specific and should be interpreted with caution [10,11]. Accumulating evidence indicates that not all HTLV-1-infected CD4^+^ T cells contribute equally to HAM, and that specific cellular subsets play a central role in viral persistence and inflammation. Among these, CD4^+^CD25^+^CCR4^+^ T cells are significantly increased in PBMCs from HAM patients [12] and display a Th1-like inflammatory phenotype characterized by the production of proinflammatory cytokines [13]. Targeting this subset has shown therapeutic benefit, as treatment with the anti-CCR4 monoclonal antibody mogamulizumab reduces the number of HTLV-1-infected cells and inflammatory markers in HAM patients [14].

In addition to CCR4^+^ T cells, an increase in CD4^+^CD45RA^−^Foxp3^low^ T cells has been reported in HAM patients [15]. Foxp3 is a key transcription factor for regulatory T cells (Tregs), and its expression in HTLV-1-infected cells is thought to be induced by the viral protein HBZ. Analyses of HBZ transgenic mice have shown that HBZ can induce Foxp3 expression in CD4^+^ T cells, although this expression is unstable [16]. In HAM patients, Tregs exhibited reduced Foxp3 expression, accompanied by impaired suppressive functions [17]. Based on these patient-derived observations, it is plausible that Foxp3^low^ Treg-derived cells contribute to the immune dysregulation characteristic of HAM.

These pathogenic CD4^+^ T cell subsets share features of sustained activation and proliferation, implying an increased demand for metabolic support. In T cells, the transferrin receptor (TfR) has been reported to regulate not only cell proliferation but also differentiation and function via iron metabolism [18,19]. TfR is selectively expressed on activated CD4^+^ T cells, whereas resting CD4^+^ T cells lack its expression. Because TfR is also present on ATLL cells, it has been explored as a potential therapeutic target [20,21]. Given that HTLV-1-infected CD4^+^ T cells are constitutively activated by viral factors such as Tax [22], we designed this study as a hypothesis-driven investigation and hypothesized that TfR expression defines a functionally distinct subset of infected CD4^+^ T cells associated with proliferation and inflammatory activity in HAM.

On the basis of this postulate, we investigated the expression of TfR on HTLV-1-infected CD4^+^ T cells isolated from asymptomatic carriers (ACs) and HAM patients. We further characterized the phenotype and functional properties of the TfR^+^ subset and examined its association with disease activity in HAM.

## 2. Materials and Methods

### 2.1. Study Subjects and Samples

Whole blood was collected, with informed consent, from healthy controls (HCs, N = 4), ACs (N = 5), and patients with HAM (N = 21). Patients with HAM were included based on a diagnosis according to WHO criteria [23]. While no formal exclusion criteria were predefined, patients were clinically evaluated and other neurological disorders were excluded. The number of subjects and collected samples, together with key demographic variables (age, sex, and treatment history), are summarized in Table 1, and individual clinical information for all subjects is provided in Supplementary Table S1. The Osame motor disability score (OMDS) was used to evaluate motor disability in patients with HAM, as previously described [24]. OMDS was assessed by the attending physician based on clinical evaluation. The assessor was not blinded to clinical information, including treatment status, but was not informed of experimental data, including TfR expression levels, at the time of evaluation. RNA-seq data from PBMCs (*n* = 13) of HAM patients were obtained from a previous study [25], while matched cerebrospinal fluid (CSF) samples (*n* = 13) were also collected but not subjected to RNA-seq. For flow cytometric analyses, the number of samples used in each experiment is indicated in the respective figure legends.

### 2.2. Isolation and Culture of PBMCs

PBMCs were isolated by density-gradient centrifugation using Lymphoprep (Serumwerk Bernburg AG, Bernburg, Germany) at 800× *g* for 20 min at room temperature and stored at −80 °C until analysis. Cells were cultured in RPMI-1640 medium supplemented with 10% fetal bovine serum (HyClone Laboratories, Logan, UT, USA), 100 U/mL penicillin (Meiji Seika Pharma, Tokyo, Japan), and 100 µg/mL streptomycin (Meiji Seika Pharma) at 37 °C in 5% CO_2_. For cytokine secretion assays, PBMCs were stimulated with 50 ng/mL phorbol 12-myristate 13-acetate and 2 µM ionomycin for 4 h in the presence of 2 µM monensin (BioLegend, San Diego, CA, USA).

### 2.3. Antibodies

The following fluorescent-conjugated antibodies were used for flow cytometric analyses: PE-Cy7 anti-human CD3 (clone SK7), APC-Cy7 anti-human CD4 (clone RPA-T4), Alexa Fluor 647 anti-human CD71 (clone CY1G4), PE-Cy7 anti-human CD152 (clone BNI3), BV510 anti-human Ki-67 (clone Ki-67), BV421 anti-human IL-10 (clone JES3-9D7), and Alexa Fluor 488 anti-human IFN-Ƴ (clone 4S.B3), which were purchased from BioLegend. The corresponding Alexa Fluor 647 Mouse IgG2a κ isotype control (clone MOPC-173, BioLegend) was used for CD71 staining. PE-Cy5.5 anti-human CD8 (clone SK1) and BB700 anti-human Foxp3 (clone 236A/E7) were purchased from BD Biosciences (San Jose, CA, USA). Super Bright 436 anti-human CD25 (clone BC96) was purchased from Thermo Fisher Scientific (Waltham, MA, USA). Biotinylated anti-human CADM1 (clone 3E1) was purchased from MBL (Nagoya, Japan). PE-conjugated streptavidin was purchased from BioLegend.

### 2.4. Flow Cytometric Analyses

**Figure 1:**
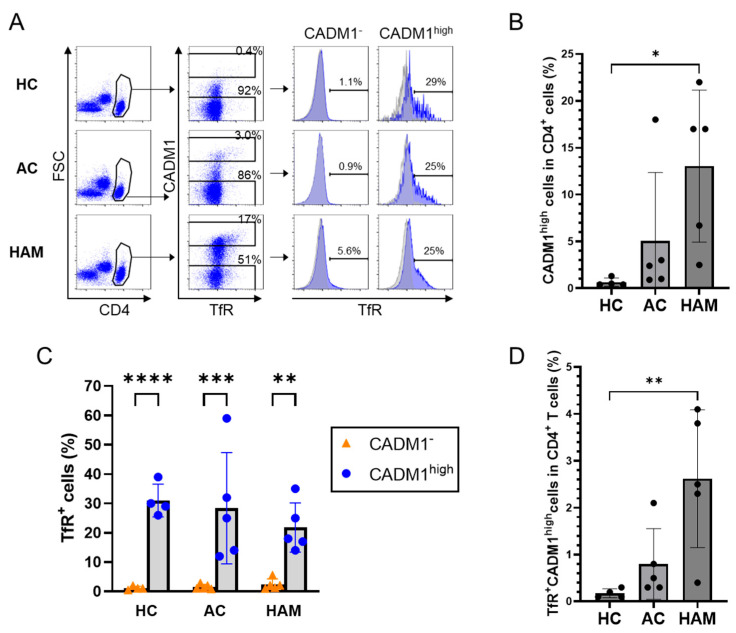
Expansion of transferrin receptor (TfR)-expressing human T-cell leukemia virus type 1 (HTLV-1)-infected CD4^+^ T cells in patients with HTLV-1-associated myelopathy (HAM). (**A**) Representative flow cytometry plots showing the expression of CADM1 and TfR in CD4^+^ T cells from peripheral blood mononuclear cells (PBMCs) of healthy controls (HCs, *n* = 4), asymptomatic carriers (ACs, *n* = 5), and HAM patients (*n* = 5). Histograms are overlaid, with TfR shown in blue and the corresponding isotype control shown in gray. (**B**) Frequencies of CADM1^high^ cells within CD4^+^ T cells based on the data in (**A**). (**C**) Frequencies of TfR^+^ cells within CADM1^high^ and CADM1^−^ CD4^+^ T cells based on the data in (**A**). (**D**) Frequencies of TfR^+^ CADM1^high^ cells within CD4^+^ T cells based on the data in (**A**). Data are presented as means ± SEM. * *p* < 0.05, ** *p* < 0.01, *** *p* < 0.001, **** *p* < 0.0001.

For intracellular cytokine staining, cells were stained using a Zombie Aqua Fixable Viability Kit (BioLegend) at room temperature for 10 min to exclude dead cell. Cells were then treated with Clear Back (MBL) at room temperature for 5 min to inhibit antibody binding to Fc receptors. Subsequently, the cells were incubated with cell surface antibodies for 15 min at 4 °C. Following cell surface staining, cells were fixed and permeabilized using the Foxp3/Transcription Factor Fixation/Permeabilization Concentrate and Diluent (Thermo Fisher Scientific) for Foxp3 and CTLA-4 staining, or the Cyto-Fast Fix/Perm Buffer Set (BioLegend) for intracellular cytokine staining, according to the manufacturers’ instructions. After permeabilization, the cells were stained with intracellular antibodies for 30 min at room temperature. Cells were washed and analyzed using a FACS Canto II (BD Biosciences), and data were analyzed using FlowJo software v10.9.0 (BD Biosciences). For gating analysis, CADM1^high^ cells were defined by setting a threshold such that the CADM1^low^ population observed in HCs, representing non-infected cells, was excluded from the gate (Figure 1A).

### 2.5. HTLV-1-Infected CD4^+^ T-Cell Sorting

HTLV-1-infected CD4^+^ T cells were isolated using the HAS-flow method described previously [25]. Briefly, PBMCs were stained with antibodies against CADM1, CD7, CD3, CD4, and CD14, followed by incubation with streptavidin–PE. Dead cells were excluded by propidium iodide staining, and CD3^+^CD4^+^CADM1^+^CD7^−^CD14^−^ cells were sorted using a FACSAria II SORP (BD Biosciences).

### 2.6. RNA Sequencing and Analysis

RNA sequencing was performed as previously described [25]. Briefly, total RNA was extracted from sorted cells using TRIzol reagent (Thermo Fisher Scientific). RNA integrity was assessed with an Agilent 2100 Bioanalyzer (Agilent Technologies, Santa Clara, CA, USA) and 20 ng of RNA with an RNA integrity number (RIN) > 7 was used for library preparation. Library construction and sequencing were performed by GeneWiz (Azenta Life Sciences, South Plainfield, NJ, USA). Indexed libraries based on rRNA depletion were pooled and sequenced on an Illumina NovaSeq platform (Illumina, San Diego, CA, USA) to generate 2 × 150 bp paired-end reads. Adapter trimming and quality filtering (Q < 20) were performed using Trimmomatic (v0.30) and clean reads were aligned to the reference genome with Hisat2 (v2.0.1). Gene expression levels were compared using transcripts per million (TPM).

### 2.7. Measurement of CXCL10 and Neopterin in CSF

The measurement of CXCL10 and neopterin was performed as reported previously [25]. CSF neopterin concentrations were determined by high-performance liquid chromatography (SRL Inc., Tokyo, Japan) and CXCL10 levels were assessed using a cytometric bead array (BD Biosciences).

### 2.8. Statistical Analysis

Statistical analyses were performed with GraphPad Prism version 10 (GraphPad Software, Boston, MA, USA). The statistical results are expressed as the mean ± standard error of the mean (SEM) (*n* = 3–5). For multiple group comparisons, one-way ANOVA was followed by Dunn’s multiple comparisons test and two-way ANOVA was followed by Tukey’s multiple comparisons test. The Pearson correlation coefficient (Pearson’s r) was calculated to analyze correlations between TfR gene expression and each of the following: neopterin concentration, CXCL10 concentration, and the Osame motor disability score (OMDS). *p*-values < 0.05 were considered statistically significant.

## 3. Results

### 3.1. Expansion of TfR-Expressing HTLV-1-Infected CD4^+^ T Cells in Patients with HAM

To investigate TfR expression in HTLV-1-infected CD4^+^ T cells, we used CADM1 as a surrogate marker to identify infected cells and analyzed the frequency of CADM1^high^ cells in PBMCs from HCs, ACs, and patients with HAM. The clinical characteristics of the study subjects are summarized in Table 1.

CD4^+^ T cells from HCs exhibited a very low frequency of CADM1^high^ cells, whereas a markedly higher frequency of CADM1^high^ cells was observed in patients with HAM (Figure 1A,B). CADM1^high^CD4^+^ T cells showed a higher frequency of TfR expression compared with CADM1^−^CD4^+^ T cells (Figure 1A,C), indicating that TfR expression was selectively enriched in HTLV-1-infected cells relative to uninfected cells. Notably, a small fraction of TfR^+^ cells was also detected within the CADM1^−^ subset (range: 0.5–5.6%, mean: 1.2–2.4% across groups), indicating that while TfR is predominantly associated with infected cells, a minor CADM1-independent TfR^+^ population exists. Within the CADM1^high^ population, however, TfR was not uniformly expressed across all HTLV-1-infected cells, but was restricted to a specific subset. Notably, the proportion of TfR-positive cells within the HTLV-1-infected (CADM1^high^) population was comparable between ACs and patients with HAM (Figure 1A,C). In contrast, because the overall frequency of HTLV-1-infected CD4^+^ T cells was increased in the HAM group, the proportion of TfR^+^CADM1^high^ cells among total CD4^+^ T cells was significantly elevated in patients with HAM (Figure 1D). These findings indicate that TfR is characteristically expressed in a subset of HTLV-1-infected CD4^+^ T cells and is present at comparable frequencies among infected cells in ACs and patients with HAM, whereas the overall frequency of TfR^+^ infected cells was increased in HAM patients because of the expansion of the infected cell population.

### 3.2. The TfR^+^ Subset Identifies Proliferative Treg-like Cells Within HTLV-1-Infected CD4^+^ T Cells

**Figure 2:**
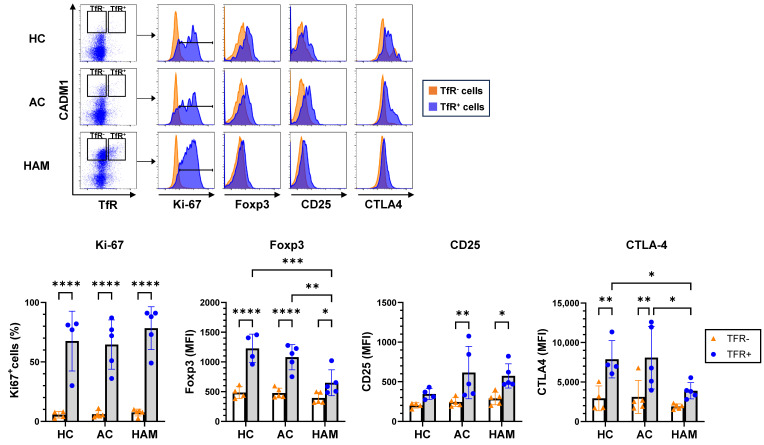
The TfR^+^ subset is identified as proliferative Treg-like cells within HTLV-1-infected CD4^+^ T cells. Representative flow cytometry plots and corresponding quantitative analyses of PBMCs from healthy controls (HCs, *n* = 4), asymptomatic carriers (ACs, *n* = 5), and patients with HAM (*n* = 5) showing the frequency of Ki-67^+^ cells and the mean fluorescence intensity (MFI) of Foxp3, CD25, and CTLA-4 in the TfR^+^ and TfR^−^ subsets of CADM1^high^CD4^+^ T cells. Data are presented as means ± SEM. * *p* < 0.05, ** *p* < 0.01, *** *p* < 0.001, **** *p* < 0.0001.

To characterize the phenotypic properties of the TfR^+^ subset within HTLV-1-infected CD4^+^ T cells, we performed a comparative analysis of TfR^+^ and TfR^−^ subsets among CADM1^high^CD4^+^ T cells. As a result, the TfR^+^ subset exhibited a significantly higher frequency of Ki67 expression, a marker of cell proliferation, compared with the TfR^−^ subset (Figure 2). In ACs, Foxp3, a key transcription factor and marker of Tregs, was expressed at higher levels in TfR^+^ than in TfR^−^ CADM1^high^CD4^+^ T cells (TfR^+^: 1079 ± 211 [95% CI: 817–1341], TfR^−^: 476 ± 83 [95% CI: 374–579]; fold difference: 2.3). In HAM patients, Foxp3 MFI in TfR^+^ cells was higher than in TfR^−^ cells (TfR^+^: 650 ± 218 [95% CI: 380–920], TfR^−^: 392 ± 93 [95% CI: 277–507]; fold difference: 1.7). However, Foxp3 MFI in TfR^+^ cells from HAM patients was substantially lower than that in TfR^+^ cells from ACs. Other Treg-associated markers, including CD25 and CTLA-4, were expressed at higher levels in TfR^+^ than in TfR^−^ CADM1^high^CD4^+^ T cells in both ACs and HAM patients. However, CTLA-4 expression in TfR^+^ cells from patients with HAM was lower than that in TfR^+^ cells from ACs (Figure 2). Notably, although present at a much lower frequency, TfR^+^ cells within the CADM1^−^ subset exhibited a Foxp3^+^Ki-67^+^ phenotype similar to that of TfR^+^ cells in the CADM1^high^ population (Supplement Figure S1), consistent with a regulatory T cell-like or activated T cell phenotype. These findings indicate that TfR^+^ HTLV-1-infected CD4^+^ T cells represent a proliferative Treg-like population, and that Foxp3 and CTLA-4 expression in this subset is reduced in patients with HAM.

### 3.3. TfR^+^ Subset of HTLV-1-Infected CD4^+^ T Cells Exhibits IFN-γ Production Capacity

**Figure 3:**
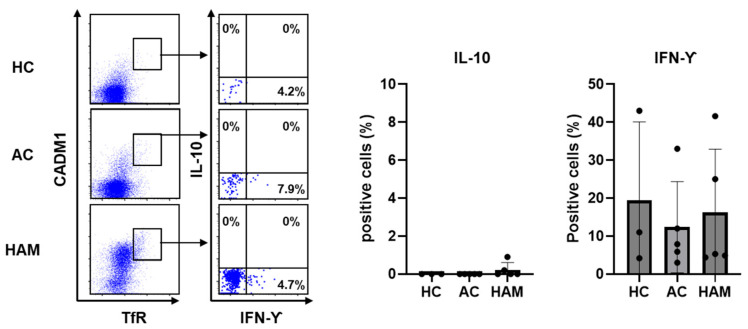
The TfR^+^ subset of HTLV-1-infected CD4^+^ T cells exhibits IFN-γ production capacity. PBMCs from healthy controls (HCs, *n* = 3), asymptomatic carriers (ACs, *n* = 5), and patients with HAM (*n* = 5) were stimulated with 50 ng/mL phorbol 12-myristate 13-acetate (PMA) and 2 µM ionomycin for 4 h. The intracellular expression of IFN-Ƴ and IL-10 in the TfR^+^ subset of CADM1^high^CD4^+^ T cells was analyzed by flow cytometry. No statistically significant differences were observed.

Previous studies have reported that Treg populations with reduced Foxp3 expression fail to fully maintain their immunosuppressive functions [17] and can acquire proinflammatory features, including the ability to produce IFN-γ [26]. Based on this, we analyzed cytokine production to further characterize the functional properties of the TfR^+^ subset within CADM1^high^CD4^+^ T cells derived from ACs and individuals with HAM. The immunosuppressive cytokine IL-10 was not detected in this subset in any group. In contrast, the proinflammatory cytokine IFN-γ was produced in all groups. In HCs, CADM1^high^ CD4^+^ T cells were present at very low frequencies; although IFN-γ production was observed, these samples were included in the statistical analysis to allow for group comparisons; however, quantitative evaluation was limited due to their low abundance, and the results should be interpreted with caution. Kruskal–Wallis analysis revealed no significant differences in IFN-γ production among HCs, ACs, and patients with HAM (*p* > 0.05). In AC and HAM groups, IFN-γ production was consistently observed, and no statistically significant difference was detected between these groups (Figure 3). These findings suggest that the TfR^+^ subset of HTLV-1-infected CD4^+^ T cells is capable of producing IFN-γ irrespective of disease status.

### 3.4. TfR Expression in HTLV-1-Infected CD4^+^ T Cells Is Associated with Inflammation and Disease Severity in HAM

**Figure 4:**
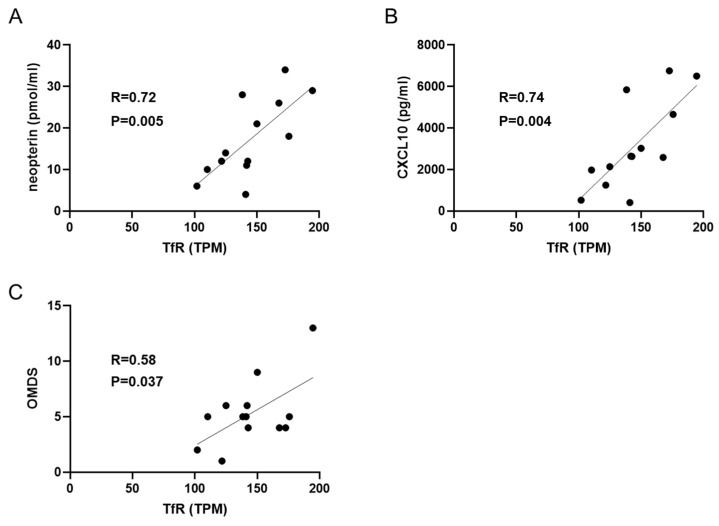
TfR expression in HTLV-1-infected CD4^+^ T cells is associated with inflammation and disease severity in HAM. Pearson correlation coefficients were calculated to assess the relationship between the expression level of the TfR gene in CADM1^high^CD7^+^CD4^+^ T cells from PBMCs, measured by RNA-seq in Transcripts Per Million (TPM), and (**A**) neopterin or (**B**) CXCL10 concentrations in the cerebrospinal fluid (CSF), as well as (**C**) the Osame motor disability score (OMDS) in patients with HAM (*n* = 13). *p*-values < 0.05 were considered statistically significant.

Given the IFN-γ production capacity of the TfR^+^ subset, we investigated whether this population was associated with inflammatory status and disease severity in HAM. Using HAS-Flow analysis [27], TfR gene expression was analyzed in D-fraction cells, defined as the population of HTLV-1-infected CD4^+^ T cells, which were isolated from the PBMCs of patients with HAM. TfR gene expression showed positive correlations with the concentrations of the inflammatory markers neopterin (*r* = 0.72, 95% CI: 0.28–0.91; *p* = 0.0054; *n* = 13) and CXCL10 (*r* = 0.74, 95% CI: 0.31–0.92; *p* = 0.0040; *n* = 13) in the CSF (Figure 4A,B). In addition, TfR expression was positively correlated with the OMDS (*r* = 0.58, 95% CI: 0.04–0.86; *p* = 0.0371; *n* = 13), an established clinical severity index for HAM (Figure 4C); however, the relatively small sample size warrants cautious interpretation due to increased sensitivity to individual data points. These findings suggest that TfR expression in HTLV-1-infected CD4^+^ T cells may be associated with central nervous system inflammation and disease severity in HAM. Thus, TfR expression could reflect the presence of HTLV-1-infected CD4^+^ T cells with an altered Treg-like phenotype in clinically relevant inflammatory settings.

## 4. Discussion

In this study, we demonstrated that TfR expression defines a distinct subset of HTLV-1-infected CD4^+^ T cells within the CADM1^high^ population. Across multiple analyses, TfR^+^ cells exhibited a proliferative phenotype characterized by high Ki-67 expression, and displayed Treg-like features based on Foxp3 and CTLA-4 expression patterns while also potentially expressing inflammatory molecules. These observations suggest that TfR marks a subset with Treg-like and proliferative features that is potentially relevant to immune regulation in HAM.

Importantly, TfR expression was only observed in a subset of CADM1^high^ cells, indicating substantial heterogeneity within the HTLV-1-infected CD4^+^ T-cell population. One possible explanation is that TfR marks an activation state induced by transient and stochastic expression of Tax [28]. Supporting this idea, TfR^+^ cells were enriched for Ki-67-positive cells, consistent with the known role of TfR in iron uptake during cell proliferation. In addition, Foxp3 can be transiently upregulated upon T-cell activation [29], suggesting that Foxp3 expression in TfR^+^ cells may not exclusively indicate stable Treg lineage commitment. Nevertheless, this activation-dependent mechanism alone may not fully explain the disease-associated reduction in Foxp3 observed in HAM patients.

An alternative explanation is that TfR^+^ cells may represent a Treg or Treg-like subset intrinsically present within the infected cell population. HTLV-1 may infect regulatory T cells, raising the possibility that a fraction of infected cells originates from this subset. Regulatory T cells are known to exhibit higher proliferative capacity [29,30] and elevated TfR expression, consistent with our observations. In addition, the viral protein HBZ has been reported to induce Foxp3 expression, thereby promoting Treg-like phenotypes. Heterogeneity in Foxp3 expression induced by HBZ may reflect differences in HBZ expression levels or variations in the local cytokine milieu. Taken together, the characteristics of TfR^+^ cells likely reflect the intrinsic properties of this proliferative Treg-like subset rather than simple activation.

TfR^+^ cells produced IFN-γ even in ACs, suggesting that this subset has potential proinflammatory activity regardless of disease status. This raises the question of why ACs do not develop HAM despite the presence of these cells. One possibility is that the pathogenic impact of TfR^+^ cells may depend on their abundance rather than their mere presence. Consistent with this, we observed a trend toward increased frequency of TfR^+^ cells in HAM patients compared with ACs, which may suggest that their accumulation could contribute to disease progression.

Another possibility is that, in ACs, TfR^+^ cells may maintain regulatory function via Foxp3, whereas in HAM, inflammatory conditions could potentially reduce Foxp3 expression and impair suppressive activity. This hypothesis is consistent with previous reports suggesting reduced Foxp3 expression and impaired Treg function in HAM patients [17]. Based on these findings, it is plausible that suppression of inflammatory HTLV-1-infected cell proliferation and proinflammatory cytokine production may be insufficient in HAM, and CTL responses against infected cells may be released from suppression, potentially enhancing inflammation. These changes could contribute to chronic inflammation; however, this remains a hypothesis that requires experimental validation.

Although TfR^+^ cells were enriched in CADM1^high^ populations, a small fraction was also present within CADM1^−^ cells. These CADM1^−^ TfR^+^ cells displayed similar proliferative and Foxp3^+^ phenotypes, suggesting that TfR expression may reflect activation or differentiation states rather than being strictly infection-specific.

TfR regulates intracellular iron metabolism, thereby modulating mitochondrial metabolism, which in turn shapes Foxp3 expression and Treg function [18]. In contrast, in systemic lupus erythematosus, TfR is critically required for Th1 cells, whereas iron overload exacerbates mitochondrial dysfunction and oxidative stress, contributing to impaired Treg function [19]. These observations suggest that iron metabolism may have a dual effect on Treg biology. In the present study, TfR expression levels in HTLV-1-infected CD4^+^ T cells were found to correlate with neopterin and CXCL10 levels, and OMDSs. This association suggests that variations in TfR expression may influence Treg function and inflammatory responses, but caution is warranted in interpreting these results.

Several limitations of this study should be acknowledged. First, sample sizes were limited in some analyses, reducing statistical power, and therefore the findings should be interpreted with caution and require validation in larger cohorts. Second, suppressive function of TfR^+^ cells was not directly assessed. Third, potential overlap with other T-cell subsets, such as CCR4^+^ cells, was not examined. Additionally, cytokine production was assessed under strong PMA/ionomycin stimulation, which may not fully reflect physiological TCR-mediated activation. Finally, while CADM1 was used as a marker of HTLV-1 infection, further validation using viral proteins such as Tax or p19 remains to be addressed in future studies.

## 5. Conclusions

This study identified TfR as a potential marker of a Treg-like subset within HTLV-1-infected CD4^+^ T cells isolated from HAM patients. TfR^+^CADM1^high^CD4^+^ T cells were characterized by reduced Foxp3 expression, increased proliferative features, and IFN-γ-producing capacity, suggesting a possible shift toward a proinflammatory phenotype. Moreover, the association between TfR gene expression and clinical markers of disease activity indicates that TfR may represent a candidate biomarker and may also provide a rationale for future studies exploring therapeutic strategies targeting this pathway in HAM.

## Figures and Tables

**Table 1:** Clinical characteristics of study participants and sample distribution.

Group	*n* (Subjects)	PBMCs (*n*)	CSF (*n*)	Age (Years)	Sex (M/F)	Treatment (%)
HC	4	4	0	53 (45–61)	4/0	NA
AC	5	5	0	71 (55–80)	1/4	NA
HAM	21	21	13	66 (43–84)	6/15	76%

HC, healthy control; AC, asymptomatic carrier; HAM, HTLV-1-associated myelopathy. PBMCs, peripheral blood mononuclear cells; CSF, cerebrospinal fluid; NA, not applicable. Age is presented as median (range). Treatment indicates patients receiving immunomodulatory therapy at the time of sampling.

## Data Availability

The datasets analyzed in this study were previously published and are available from the National Bioscience Database Center Human Database under accession numbers JGAS000835, JGAS000301, and JGAS000553 [25]. In the present study, only the TPM values of the transferrin receptor gene (*TFRC*) derived from the RNA-seq data were used.

## Supplementary Materials

The following supporting information can be downloaded at: https://www.mdpi.com/article/10.3390/pathogens15040450/s1, Supplementary Table S1. Clinical and sample characteristics of individual study participants, including availability of PBMC and CSF samples, and RNA-seq and flow cytometry analysis; Supplementary Figure S1. The TfR^+^ subset in CADM1^−^CD4^+^ T cells shows proliferative and Treg-like features. Representative flow cytometry plots and corresponding quantitative analyses of PBMCs from healthy controls (HCs, *n* = 4), asymptomatic carriers (ACs, *n* = 5), and patients with HAM (*n* = 5) showing the frequency of Ki-67^+^ cells and the mean fluorescence intensity (MFI) of Foxp3, CD25, and CTLA-4 in the TfR^+^ and TfR^−^ subsets of CADM1^−^CD4^+^ T cells. Data are presented as means ± SEM. * *p* < 0.05, ** *p* < 0.01, *** *p* < 0.001, **** *p* < 0.0001.

## Abbreviations

The following abbreviations are used in this manuscript:

HTLV-1	Human T-cell leukemia virus type 1
ATLL	Adult T-cell leukemia/lymphoma
HAM/TSP	HTLV-1-associated myelopathy/tropical spastic paraparesis
PBMCs	Peripheral blood mononuclear cells
CADM1	Cell adhesion molecule 1
Tregs	Regulatory T cells
TfR	Transferrin receptor
ACs	Asymptomatic carriers
HCs	Healthy controls
TPM	Transcripts per million
CSF	Cerebrospinal fluid
OMDS	Osame motor disability score
