# Supplementary material for: Transferrin Receptor Marks a Foxp3-Low Treg-like Inflammatory T Cell Subset Associated with Disease Severity in HAM/TSP
Source: Pathogens. 2026 Apr 21;15(4):450. doi: 10.3390/pathogens15040450 (PMC13118373; doi:10.3390/pathogens15040450)
Supplement: Supplementary file 1 [file pathogens-15-00450-s001.zip › pathogens-4206275-supplementary.pdf]

Supplementary Figure S1

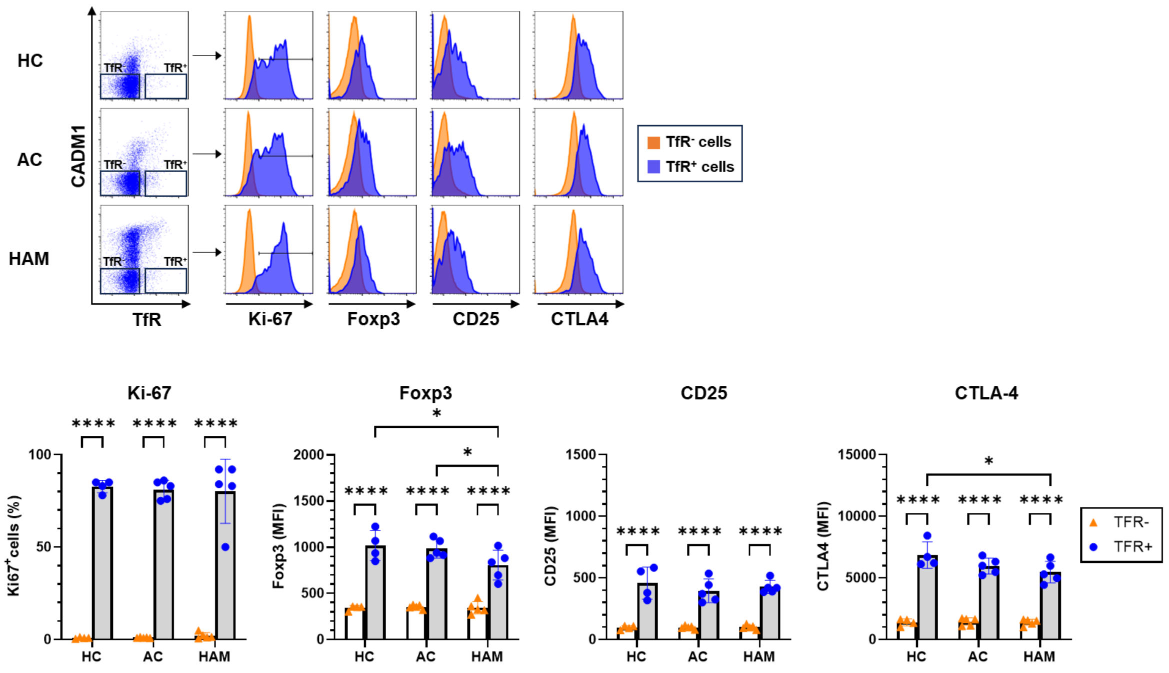

Supplementary Figure S1. The TfR⁺ subset in CADM1⁻CD4⁺ T cells shows proliferative and Treg-like features. Representative flow cytometry plots and corresponding quantitative analyses of PBMCs from healthy controls (HCs, $n = 4$), asymptomatic carriers (ACs, $n = 5$), and patients with HAM ($n = 5$) show the frequency of Ki-67⁺ cells and the mean fluorescence intensity (MFI) of Foxp3, CD25, and CTLA-4 in the TfR⁺ and TfR⁻ subsets of CADM1⁻CD4⁺ T cells. Data are presented as means ± SEM. *p < 0.05, **p < 0.01, ***p < 0.001, ****p < 0.0001.

Supplementary Table S1. Clinical and sample characteristics of individual study participants, including availability of PBMCs, CSF, RNA-seq, and flow cytometry analyses.

ID	Group	Age (years)	Sex	Treatment	PBMC	CSF	OMDS (score)	RNA-seq	Flow cytometry (phenotype)	Flow cytometry (cytokines)
HC01	HC	45	M	NA	Yes	NA	NA	No	YES	YES
HC02	HC	48	M	NA	Yes	NA	NA	No	YES	No
HC03	HC	57	M	NA	Yes	NA	NA	No	YES	YES
HC04	HC	61	M	NA	Yes	NA	NA	No	YES	YES
CA01	AC	55	F	NA	Yes	NA	NA	No	YES	YES
CA02	AC	80	F	NA	Yes	NA	NA	No	YES	YES
CA03	AC	71	F	NA	Yes	NA	NA	No	YES	YES
CA04	AC	64	F	NA	Yes	NA	NA	No	YES	YES
CA05	AC	71	M	NA	Yes	NA	NA	No	YES	YES
HAM29	HAM	59	F	No	Yes	No	7	No	No	YES
HAM32	HAM	74	F	No	Yes	No	6	No	YES	YES
HAM33	HAM	74	M	Yes	Yes	No	7	No	YES	YES
HAM34	HAM	64	M	No	Yes	No	1	No	YES	No
HAM35	HAM	71	F	Yes	Yes	No	9	No	YES	No
HAM36	HAM	70	F	Yes	Yes	No	5	No	YES	No
HAM40	HAM	74	F	No	Yes	No	7	No	No	YES
HAM47	HAM	60	M	Yes	Yes	No	9	No	No	YES
HAM050	HAM	75	M	Yes	Yes	Yes	13	YES	No	No
HAM064	HAM	71	M	Yes	Yes	Yes	4	YES	No	No
HAM366	HAM	54	F	Yes	Yes	Yes	5	YES	No	No
HAM160	HAM	72	F	Yes	Yes	Yes	5	YES	No	No
HAM330	HAM	84	F	Yes	Yes	Yes	6	YES	No	No
HAM258	HAM	65	F	No	Yes	Yes	4	YES	No	No
HAM400	HAM	44	F	Yes	Yes	Yes	5	YES	No	No
HAM349	HAM	43	F	Yes	Yes	Yes	9	YES	No	No
HAM094	HAM	65	M	Yes	Yes	Yes	1	YES	No	No
HAM179	HAM	56	F	Yes	Yes	Yes	5	YES	No	No
HAM065	HAM	67	F	Yes	Yes	Yes	6	YES	No	No
HAM464	HAM	66	F	Yes	Yes	Yes	4	YES	No	No
HAM408	HAM	66	F	Yes	Yes	Yes	2	YES	No	No

HC, healthy controls; AC, asymptomatic carriers; HAM, HTLV-1–associated myelopathy.
NA, not applicable; PBMC, peripheral blood mononuclear cells; CSF, cerebrospinal fluid.
OMDS: Osame Motor Disability Score (0–13)
Treatment indicates patients receiving immunomodulatory therapy at the time of sampling.
RNA-seq: RNA sequencing analysis available
Flow cytometry (phenotype / cytokines): Flow cytometry analysis for cellular phenotype or cytokine production
